# Leading With LARCs in Nigeria: The Stars Are Aligned to Expand Effective Family Planning Services Decisively

**DOI:** 10.9745/GHSP-D-16-00135

**Published:** 2016-06-20

**Authors:** James D Shelton, Clea Finkle

**Affiliations:** a Global Health: Science and Practice, Editor-in-Chief, Washington, DC, USA; b Bill & Melinda Gates Foundation, Seattle, WA, USA

## Abstract

Despite years of family planning effort in Nigeria, the modern contraceptive prevalence (mCPR) has reached only 10%. Yet a few recent seminal, well-executed programs have been outstandingly successful providing long-acting reversible contraceptives (LARCs)—both in the public and private sector, and in the North and South. Remarkably, the LARCs they provided were equivalent to 2% mCPR in 2015 alone.

Accordingly, we advocate markedly increased support for: (1) private-sector approaches such as social franchising, particularly in the South, (2) mobile outreach, and (3) support to public clinical facilities, including expanding access through community health extension workers (CHEWs), particularly in the North. Success will require system support, quality, and concerted engagement from a variety of partners including the Government of Nigeria.

Without significant progress in Nigeria, the global FP2020 goal appears unattainable. Fortunately, leading with LARCs along with wide choice of other methods provides a clear avenue for success.

## THE VIRTUES OF LARCS—NIGERIA IS NO EXCEPTION

Provision of long-acting reversible contraceptives (LARCs)—IUDs and implants—has been highly effective, perhaps even revolutionary in the United States, in preventing unintended pregnancy, particularly with adolescents.^1^ No single method, or even 2 methods, can satisfy the diverse needs of all clients, and clients must have good choice and access to a wide variety of methods. Yet those same attributes that have made LARCs so popular in the United States—very high effectiveness, long duration of action, independence of the sex act, generally manageable side effects, potential for use without partner knowledge, and in the case of implants no need for a pelvic exam—appear to have wide appeal in Nigeria. Still, success with LARCs requires more than simple availability of the products. LARCs require service delivery approaches with a higher degree of wherewithal and quality than for shorter-acting methods. But as described below, such LARC-enabling approaches are beginning to thrive in Nigeria.

LARCs appear to have wide appeal in Nigeria.

## COMPELLING NEED

Nigeria looms large in need for family planning. Its current 186 million population is projected to grow to almost 400 million by 2050, a mere 34 years from now.^2^ Its maternal mortality ratio of 576 per 100,000 live births^3^ is among the highest in the world—and far off the Millennium Development Goal (MDG) target of 300. Nigeria also performs poorly on most other health and development indicators, including infant mortality (69 per 1,000 live births),^3^ poverty (62%),^4^ and female literacy (50%).^5^ Moreover, there are marked health disparities between the richest and poorest.^3^

## DAUNTING CHALLENGES IN FAMILY PLANNING

Thus far, Nigeria has been rather resistant to family planning efforts. Between 2008 and 2013, the modern contraceptive prevalence rate (mCPR) remained at a mere 10%.^2^ And that use was dominated by short-acting methods. Reported ideal family size is 6.5 children. The health system itself is highly challenged,^6^ and the decentralized federal structure that delegates major functions to its 36 states and the Federal Capital of Abuja as well as to over 770 local government areas (LGAs) makes broad support and management unwieldy. Most people seek health services from a patchwork of private health care providers. Unfounded rumors about contraceptive methods are believed to be widespread. And women’s knowledge about contraception is limited, especially about LARCs. In the 2013 Demographic and Health Survey, only 25% of married women had even heard of implants and 32% of IUDs.^3^ Whereas modern contraceptive use in the more rural, less prosperous, and less literate North East and North West states is below 4%, levels are significantly higher in the southern zones, especially in the South West where contraceptive rates in 7 states range from 21% to 32%.

## YET REMARKABLY POSITIVE DEVELOPMENTS

Despite this difficult backdrop, a diverse set of recent developments provide compelling evidence that family planning efforts are beginning to make significant headway in the country.

- Between 2008 and 2013, 6 states showed gains of 5 to 12 percentage points in modern contraceptive use.^3^
- The Nigerian Urban Reproductive Health Initiative (NURHI) in 4 urban areas increased contraceptive prevalence an average of 11% in 5 years by combining substantial demand- and supply-side efforts, including highly accessible mobile “outreach” services. Over 40% of those gains came from increased use of LARCs (mostly implants). Misconceptions about contraceptives declined markedly and intent to use contraception increased significantly.^7^^,^^8^
- As described in an article in this issue of GHSP,^9^ an immediate postpartum, small-scale initiative with private providers in several states found that 41% of eligible women who delivered in those facilities received immediate insertion of an IUD—evidence that IUDs can be a popular choice among postpartum women.
- Following the 2012 London Summit on Family Planning, the Government of Nigeria articulated more prominent support for family planning including a detailed and cogent national family planning blueprint with ultra-ambitious objectives,^10^ although actual financial resources provided so far have been limited. Many states have followed suit, with state-level costed implementation plans (CIPs). Some positive policy reforms have also occurred, notably allowing a basic-level cadre of service health worker—the community health extension worker (CHEW)—to provide implants and IUDs. Additional reforms have included making contraceptives free in public-sector clinics and reforming the nurse/midwife training curriculum to make it proficiency-based and supported by rigorous supervision.

IUDs can be a popular choice among postpartum women

### Outstanding Successes With LARCs Specifically

A range of programmatic approaches in both the private and public sector emphasizing access and quality have achieved strikingly high provision of LARCs in Nigeria. Most are relatively recent:

- The Marie Stopes Nigeria “BlueStar” social franchising program, launched in 2012, supports more than 300 private-sector providers, especially in the South, of whom about 70% are midwives. They provided a notably large number of women—more than 65,000—with contraception, especially implants, in 2015 (Table).
- The Society for Family Health’s Healthy Family Network social franchising program, also with more than 300 providers, has likewise been successful, although in its case more with the IUD (Table).
- A more recent entry to Nigeria, DKT International also gives support to private providers, particularly in the South. Its model consists mainly of marketing contraceptive products to providers and distributors, without the networking and other components of social franchising. LARC sales in 2015—at more than 100,000—are also striking (Table).
- Marie Stopes Nigeria deploys “mobile outreach” teams in the North. Such mobile outreach typically occurs in collaboration with the public sector and in public-sector sites. Remarkably, with only 8 outreach teams, they provided some 63,000 clients with LARCs in 2015, primarily implants (Table).
- Marie Stopes Nigeria also began the Family Health Plus initiative in 2014. It works with public-sector providers at the state level, now expanded to 20 states, emphasizing training, supportive supervision, and supply chain. Again, provision of LARCs to more than 250,000 clients in 2015 is highly impressive (Table).

**TABLE:** Provision of Long-Acting Reversible Contraceptive Methods by Selected Program Initiatives, Nigeria, 2015

Program	Implants	IUDs	Total LARCs
BlueStar (Marie Stopes Nigeria)	51,643	13,811	**65,454**
Healthy Family Network (SFH)	20,273	53,900	**74,173**
DKT International sales	15,967	87,600	**103,567**
Mobile outreach (Marie Stopes Nigeria)	53,786	9,200	**62,986**
Family Health Plus (Marie Stopes Nigeria)	222,705	29,686	**252,391**
**Total**	**364,374**	**194,197**	**558,571**

Abbreviations: IUDs, intrauterine devices; SFH, Society for Family Health; LARCs, long-acting reversible contraceptives. Source of data: For Marie Stopes Nigeria, personal communication with Anna Mackay, Deputy Director of SIFPO Project, Marie Stopes International; for SFH, personal communication with Peter Entonu, Associate Director of Social Franchise Unit, SFH; for DKT, sales report from December 2015.^11^

### What Is Particularly Notable About These Findings From the 5 Initiatives?

- **Sizable enough to “move” the needle.** More than 550,000 women received LARCs from just these 5 initiatives, representing over 2% of married women of reproductive age and 1.5% of all women of reproductive age in Nigeria nationally—a truly impressive proportion (Table). Of course these numbers don’t translate directly into contraceptive prevalence. For example, some women would have switched from other methods, and sales do not precisely reflect method provision. Still, the results from just these 5 projects in just 1 year should be enough to register population-level impact. And because LARCs engender high rates of satisfaction and continuation, the sustained cumulative effect over a number of years would be substantial. Some corroboration of such an impact come from the Performance Monitoring & Accountability 2020 (PMA2020) surveys in Kaduna state in the North—a state where Family Health Plus, Healthy Family Network, Marie Stopes Nigeria mobile outreach, and NURHI (as well as others) are all active. Between 2014 and 2015, the *share* of overall modern contraceptive use for implants in Kaduna increased from 16% to 28%.^12^
- **Substantial untapped “latent” demand.** While these initiatives appropriately include modest demand-side components, they mainly consist of making contraceptive services highly accessible. Thus, a significant amount of demand for modern contraception among a sizable proportion of women already exists that can be satisfied with good services and a modicum of targeted demand “activation” support.
- **LARCs are highly acceptable for women desiring contraception.** This is particularly the case for implants, but also for IUDs.
- **Promoting equity: LARC programming serves very low-income as well as higher-income clients.** For example, client exit interview data indicate that 75%, 53%, and 49% of Marie Stopes Nigeria's mobile outreach, Family Health Plus, and social franchising clients, respectively, were from *households* living on less than US$2.50 per day (personal communication with Anna Mackay, Deputy Director of SIFPO Project, Marie Stopes International).
- **Success in both the South and North.** While it is generally held that the more conservative North is highly resistant to family planning, these projects—notably, mobile outreach and the Family Health Plus public-sector support project—demonstrate that success can be achieved in the North as well.
- **Success in both the private and public sectors.** Private-sector approaches including social franchising align well with the South, which has a large concentration of small private-sector providers and is more economically advanced. But the Family Health Plus experience shows that despite the public sector’s reputation as a weak service delivery platform, working closely with state governments on public-sector service delivery can be successful.

LARCs serve both low- and high-income clients.

**Figure:**
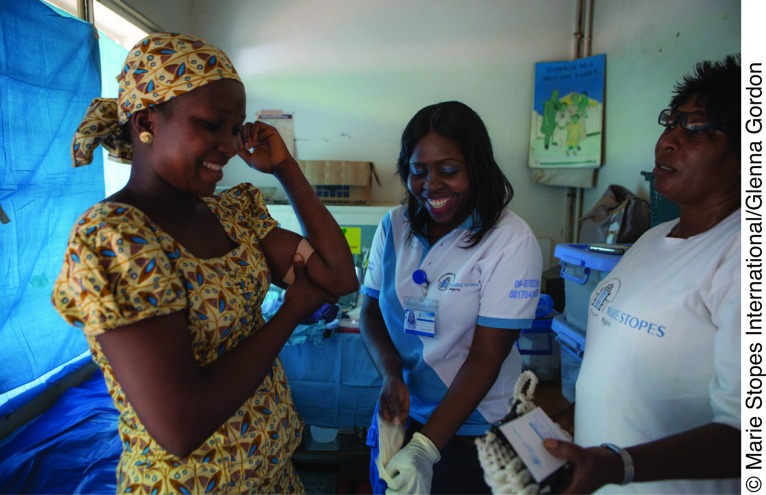
A woman in Nigeria has had a contraceptive implant inserted by providers from Marie Stopes Nigeria.

In addition to the programming described above, a number of other worthwhile initiatives are ongoing in Nigeria, including traditional social marketing and clinic-based public-sector and NGO services. We are merely highlighting some of the more prominent programs that have been highly successful with LARCs.

## WHAT NEXT: OUR PRIORITIES FOR EXPANDED FAMILY PLANNING PROGRAMMING

Our proposed overall family planning strategy for Nigeria:

**Support a diversity of service delivery approaches and wide method choice, but emphasize the private sector in the South and the public sector in the North. And prioritize service modes that include LARCs effectively.**

Our proposed strategy: Support a diversity of service delivery approaches and wide method choice, including LARCs; emphasize the private sector in the South and the public sector in the North.

Family planning is most successful when clients have a wide variety of methods to choose from, and clients can choose from a variety of service delivery sites. Accordingly, it makes strategic sense to pursue diverse programming efforts in both the public and private sector across the country, emphasizing those models that have a good track record. Recognizing that about 60% of contraceptive use nationally comes from the private sector, private/commercial-sector approaches such as social franchising are particularly well-suited for the South where of the very wide distribution of private-sector providers and the entrepreneurial climate. However, in the North, where that private-sector infrastructure is currently thin, priority should be with the public and NGO sectors. It also seems reasonable to be opportunistic by working most with states that are willing to commit more of their own resources. Since LARCs, especially implants, are so clearly popular and satisfy the needs of many clients well, it is wise to invest in service delivery modes that can provide LARCs effectively, along with a broad range of methods including injectables. Building on the successful models described above, we present some details of this strategy.

**Social franchising.** Current efforts in social franchising have been highly successful as described above, and indeed in a wide variety of other countries, and they should be expanded markedly, particularly in the South.

**Mobile outreach.** Although so far small in scale, mobile outreach has been shown to be highly effective already, particularly in the most difficult part of the country—the North and for hard-to-reach rural populations. But this proven approach should also be deployed in other areas such as urban slums and peri-urban areas generally. Notably, it was part of the highly effective urban NUHRI project in the North and South.

**Expanded provision of LARCs and injectables by CHEWs,** particularly in the public sector. Having CHEWs provide LARCs has been shown to be effective in research studies^13^ and now presents a major opportunity for mainstream service delivery expansion, given the recently liberalized policy to permit it. A first order of activity is to scale-up training and supportive supervision for CHEWs to provide LARCs, thus nurturing “dedicated providers” who, in turn, become mentors to new providers. CHEWs have many other duties, however, and some will be more motivated to provide LARCs than others. Thus, finding a way to provide good support to the most productive CHEWs is essential. Provision of injectables by CHEWs should be a complementary priority, especially in the North where CHEWs are often the main or only clinical providers.

**Social marketing, especially focusing on injectables.** The successful model DKT is following deserves considerable expansion. An additional opportunity is extending provision of injectables through the many qualified proprietary patent medicine vendors (PPMVs) in both the North and the South. Heretofore, PPMVs have not been permitted to provide injectables. However, a recent PPMV census found that over half in Northern states and about 30% in Southern states were staffed by trained professionals including nurses, pharmacists, and CHEWs.^14^ Since CHEWs are now permitted to provide injectables (and implants) in the public sector, it seems entirely reasonable that PPMVs trained as CHEWs or nurses should also be permitted to give injectables. Sayana Press, the version of DMPA delivered subcutaneously via the prefilled Uniject device, is also being introduced in Nigeria, which offers a variety of advantages and additional potential for expanded injectable use.

**Postpartum contraception, especially IUDs and implants.** The results with immediate postpartum IUDs provided by private-sector providers described above are very promising. Nationally, 36% of women deliver in facilities, with significantly higher rates of facility delivery in the South compared with the North.^3^ Especially for high-volume facilities where providers are more likely to retain skills, immediate postpartum insertion of IUDs or later provision of IUDs, implants, or other methods before discharge can be an important component of programming.^15^ Moreover, providing contraception later in the postpartum period, for example, along with immunization services, can be highly effective for reaching women during this time of very high unmet need.^16^ And mobile outreach in conjunction with child immunization services can successfully reach women in the postpartum period.

### Key System Support Priorities

In addition to the priority program approaches described above, serious attention is needed to the following system support activities.

**Demand support.** The programmatic approaches we propose benefit markedly from a healthy component of demand activation, including community engagement to promote awareness and to address specific issues such as misconceptions about contraception. In addition, broader demand support such as through mass media is a compelling priority, addressing key issues such as social norms and the health benefits of birth spacing.

**Supply chain.** Family planning cannot succeed without functional supply chains. The current variably functioning public-sector supply chain depends not only on the federal government but on states and LGA support as well. It will require considerable additional attention. One progressive innovation is the “informed push” model to help ensure good distribution, which has been successfully piloted in Nigeria.^17^^,^^18^ Private-sector distribution, such as for social franchising, can use a more commercial-sector type of supply chain approach capitalizing on the economic incentive, since participants along the chain are paying for the commodity. LARCs have the distinct advantage over shorter-acting methods that they do not require frequent resupply. On the other hand, they do requirea modest but important amount of equipment and disposable supplies such as gloves and disinfectants, which require additional supply attention.

Family planning cannot succeed without functional supply chains.

**Human resources.** The “2014 Nigeria Family Planning Blueprint” lays out the major challenges: lack of staff, lack of training, high turnover, and uneven geographic distribution in the public sector,^10^ all of which need to be addressed systematically. Progressive task shifting will help. A major advantage of private-sector providers is that many are underemployed. Thus, making good use of private-sector CHEWs and qualified PPMVs to give injectables should be a high priority.

**Policy and advocacy.** Advocacy is needed at both the federal level and the state level toward meaningful funding for family planning. Again, key policy reforms, such as permitting trained pharmacists and PPMVs to provide injectables would make a considerable difference.

**Monitoring and evaluation.** Nigeria is fortunate that PMA2020 will be expanding its annual surveys from 2 states to 7, beginning this year (personal communication with Scott Radloff, Director, PMA2020). Priority should be given to support policy makers and program managers to improve data use to enable course correction and decisions about resource allocation in those states.

**Emerging primary health care platforms.** The Government of Nigeria has recently resolved to strengthen primary health care overall,^6^ and donors such as the U.S. Agency for International Development (USAID), the Department for International Development (DFID), and the Bill & Melinda Gates Foundation are providing significant support for such platforms in selected states. This emphasis should be a good opportunity to advance family planning services, but only if sufficient resources are forthcoming and key preventive services such as family planning are given high priority. A key opportunity would be inclusion of a robust set of family planning services under the planned national health insurance scheme.^19^

## LEADING WITH LARCS - THE TIME IS NOW

The evidence is clear that family planning efforts not only can be successful—but *are* being successful—in Nigeria, especially through provision of LARCs. Nigeria is a large and complex country and ultimate success will require substantial resources, long-term attention, and a variety of approaches. Yet as we have seen, providing LARCs when done well with the proper service delivery wherewithal can be remarkably effective. Investments in these modes will go a substantial distance to meet client needs, stem unintended pregnancies, reduce maternal mortality, and support Nigeria’s national family planning goals as well as the FP2020 global goal. Success will require substantial commitment, engagement, and partnership across the board, including the Government of Nigeria. The time is right for a substantial investment in Nigeria. Do not let this compelling opportunity pass by.

## Notes

**First Published Online:** 2016 May 19
